# “Mickey Mousing” in the Brain: Motion-Sound Synesthesia and the Subcortical Substrate of Audio-Visual Integration

**DOI:** 10.3389/fnhum.2021.605166

**Published:** 2021-02-15

**Authors:** Bruno Laeng, Camilla Barthel Flaaten, Kjersti Maehlum Walle, Anne Hochkeppler, Karsten Specht

**Affiliations:** ^1^Department of Psychology, University of Oslo, Oslo, Norway; ^2^RITMO Centre for Interdisciplinary Studies in Rhythm, Time and Motion, University of Oslo, Oslo, Norway; ^3^NORMENT Centre for Research on Mental Disorders, Division of Mental Health and Addiction, University of Oslo and Oslo University Hospital, Oslo, Norway; ^4^Norwegian Institute of Public Health, Oslo, Norway; ^5^German Centre for Neurodegenerative Diseases (DZNE), Magdeburg, Germany; ^6^Department of Biological and Medical Psychology, University of Bergen, Bergen, Norway; ^7^Department of Education, UiT/The Arctic University of Norway, Tromsø, Norway; ^8^Mohn Medical Imaging and Visualization Centre, Haukeland University Hospital, Bergen, Norway

**Keywords:** synesthesia, multisensory integration, motion, sound, connectivity, small-world connectivity, superior colliculus, subcortical structures

## Abstract

Motion-sound synesthesia is characterized by illusory auditory sensations linked to the pattern and rhythms of motion (dubbed “Mickey Mousing” as in cinema) of visually experienced but soundless object, like an optical flow array, a ball bouncing or a horse galloping. In an MRI study with a group of three synesthetes and a group of eighteen control participants, we found structural changes in the brains of synesthetes in the subcortical multisensory areas of the superior and inferior colliculi. In addition, functional magnetic resonance imaging data showed activity in motion-sensitive regions, as well as temporal and occipital areas, and the cerebellum. However, the synesthetes had a higher activation within the left and right cuneus, with stronger activations when viewing optical flow stimuli. There was also a general difference in connectivity of the colliculi with the above mentioned regions between the two groups. These findings implicate low-level mechanisms within the human neuroaxis as a substrate for local connectivity and cross activity between perceptual processes that are “distant” in terms of cortical topography. The present findings underline the importance of considering the role of subcortical systems and their connectivity to multimodal regions of the cortex and they strengthen a parsimonious account of synesthesia, at the least of the visual-auditory type.

## Introduction

Synesthesia is a phenomenon where a sensed property automatically evokes the sensation of another property that is not there (Cytowic and Eagleman, 2009), either within the same modality or across modalities. For example, the Finnish composer Jean Sibelius reported to his biographer (Ekman, 1938) that when he looked at an object colored green, he would hear in his head an F major chord; the physicist Feynman (1988) reported in his autobiography that whenever he saw equations he saw them in colors (despite the alphanumeric symbols were achromatic). These representative individuals’ conditions are today called grapheme-color and music-color synesthesia, respectively. A score of studies within the last twenty years brought evidence to the validity and reliability of these phenomenological reports in terms of their effects on both behavior and neural processes (e.g., Beeli et al., 2005; Laeng et al., 2011), despite their solipsistic privacy and lack of phenomenological intersubjectivity.

### Cross-Connectivity in the Brain of Synesthetes: Cortical or Sub-Cortical Too?

Synesthesia presents an extreme, puzzling, case of the human brain’s neuroplasticity and its integration of different perceptual modalities. A leading hypothesis about the neural basis of synesthesia is that these individuals possess extra neural connectivity and interactivity across neighboring cortical areas that channel different perceptual features (Hubbard et al., 2011). A “local,” structural, account of synesthesia has received support from diffusion tensor imaging (DTI) and voxel-based morphometry (VBM) analyses of the brains of grapheme-color synesthetes (Rouw and Scholte, 2007, 2010; Jäncke et al., 2009; Weiss and Fink, 2009; van Leeuwen et al., 2011), confirming atypical connectivity and increased gray matter within neighboring cortical regions supporting the properties of synesthetically linked perceptions. As a reason for such hyper-connectivity within the synesthetic brain, it has been proposed a reduced “neural pruning” during development (Hubbard and Ramachandran, 2005) that, given the “small world” properties of cerebral connectivity, would result in enhanced cross-activity between neighboring, local, regions. This could in turn explain the augmented perceptual integration, even when one of the involved modalities is not stimulated (e.g., Sibelius’ hearing of a musical chord as green is illusory).

Recent models have proposed ways in which crossover sensory information and ‘high level’ feedback could give origin to neurally distant forms of synesthetic integration by long-range disinhibited feedback or re-entrant processing (e.g., Hubbard and Ramachandran, 2005; Neufeld et al., 2012). However, a parsimonious possibility is that the “lower” or “local” type of neural cross-activation and structural integration could play a more important role than previously considered also for forms of synesthesia that appear to depend on “higher” level, functional, integrative processes between widely separated neural regions. Generally, multisensory integration depends on the interactivity among neurons at multiple levels of the neuroaxis (Stein and Stanford, 2008), including neuronal dialogues at early stages across structures in the brainstem. Importantly, senses that appear to be distant on the cortical mantle can be adjacent and strongly inter-connected at the subcortical level. For example, animal studies clearly indicate that the both the inferior and superior colliculi in the midbrain bind visual and auditory information (Stanford et al., 2005; Gruters and Groh, 2012; for a review: Casey et al., 2012).

It seems fair to say that we know extraordinarily little about the relationship between subcortical processing in humans and synesthesia; perhaps not surprisingly, since there is a general lack of MRI research on small brain structures, especially human subcortical structures (still *terra incognita*, according to Alkemade et al., 2013). A subcortical structures” involvement has been occasionally mentioned in studies of synesthesia (e.g., the thalamus in Melero et al., 2013; or the cerebellum and thalamus, as found retrospectively using structural atlases by Rouw et al., 2011) but generally it has been either neglected or rejected (Bargary and Mitchell, 2008). Yet, a lack of focus on subcortical structures may have missed important clues to the underlying neural mechanisms of multi-modal integration phenomena. Because we know less about the functional profile of the subcortical compared to cortical structures, it complicates the understanding of cross-modal integration in general as well as synesthesia, given that we do know their importance in early multisensory perception and integration (e.g., Stein and Stanford, 2008).

We note that some previous studies on other types of synesthesia than the one investigated here (e.g., in grapheme-color synesthesia) have found global connectivity alterations (e.g., Hänggi et al., 2011; Dovern et al., 2012). One critical review of brain imaging studies of synesthesia found little overlap between brain activation studies from different neuroimaging studies (Hupé and Dojat, 2015), suggesting there can be wide individual differences in the neural substrate of synesthesia.

### Motion-Sound Synesthesia

Here, we focus on a form of synesthesia that is clearly cross-modal and characterized by visually induced auditory perceptions. In “motion-sound” synesthesia, there is a mandatory triggering of auditory rhythmical perceptions also when soundless visual stimuli that are pulsating in time are presented. A previous cognitive study (Saenz and Koch, 2008a) substantiated these illusory auditory perceptions in four synesthetes who, in a short-term memory task, revealed a substantial rhythmic-discrimination advantage in a difficult matching-to-sample task of visual pulses compared to matched controls. Their perceptual-mnemonic advantage could be straightforwardly attributed to their visuo-auditory synesthesia, which translated a difficult visual task into an easier auditory discrimination task.

These synesthetes’ phenomenological reports have in common that, whenever a visual object was seen moving (e.g., a bird flying, a ball bouncing, a horse galloping, a man running), even in silent displays, their visual perception was accompanied by characteristic sounds that matched closely the motion in speed of change, periodicity, pitch (e.g., high with upward movement). Remarkably, the concurrent acoustic illusions are characterized by modulations in roughness and flux in timbre as well as sweeping or rhythmic fluctuations in pitch. In a metaphor, the experience in the “mind’s ear” of these synesthetes seemed analogous to “mickey mousing” in movies and cartoon animations (e.g., the use of sounds or music to “reinforce an action by mimicking its rhythm exactly”; Eitan and Granot, 2006). A good example of cinematic “mickey mousing” might be Stanley Kubrick’s use of music and sounds in “*2001: A Space Odyssey*” (e.g., the optical flow scene in the “Jupiter and beyond” sequence, when Bowman sees stars streaming outside the shuttle, accompanied by a sound stream). A strong “sound flow” can be reported by these class of synesthetes when viewing an optical flow of dots (as used in previous and the present experiments) or, in everyday life, when traveling by car in a tunnel.

### The Present Study

We present here neuroanatomical evidence acquired with MRI in motion-sound synesthetes that structural changes occur between neighboring neural areas if one considers subcortical structures. We note that demonstrating a subcortical involvement in sound-motion synesthesia can shed a completely new light on the process of cross-activation in general as a fundamental principle for cross-modal synesthesia. Hence, in the present study, we scanned the brains of eighteen control participants with no form of synesthesia and of three motion-sound synesthetes while they viewed soundless animated and static visual sequences.

## Meterials and Methods

### Participants

The target participants were three synesthetes (two females, one male, mean age 33.6 ± 8) and, in addition, 18 adult female non-synesthetes as control participants (mean age 23.8 ± 2.9). Two contacted voluntarily the first author while the third was contacted via word-of-mouth. Synesthetic experiences were repeatedly probed in several encounters and synesthetes confirmed experiencing sounds for all the type of stimuli used in the experiment and in separate occasions. Two of the synesthetes declared other forms of synesthesia (i.e., calendar synesthesia, touch-sound), which were, however, not investigated. None of the synesthetes or control participants had a history of either neurological or psychiatric disorders. The control participants were recruited from the student population at the University of Bergen and Haukeland University Hospital. Handedness was determined by a modified version of the Edinburgh Inventory (Oldfield, 1971), which contained in total 15 questions, including the original items of the Edinburgh Inventory and some additional everyday tools. Items were scored as “mainly left,” “mainly right,” or “both hands.” One synesthete was left-, one right-, and one mixed-handed. Sixteen of the control participants were right-handed, one was left-handed, and one was mixed-handed. All participants gave written informed consent in accordance with the Declaration of Helsinki and local institutional guidelines. The regional ethics committee of the State Department of Health (REK) approved the study (#2014/324).

### Stimuli and Experimental Procedure

Participants were scanned with MRI while they simply viewed several moving visual stimuli through goggles. No response was required from participants other than to carefully observe the animations. There were three types of stimuli, all known to generate synesthetic sound experiences in all of the present synesthetes: (a) single geometrical object (triangles, squares, circles in different colors on a black screen) moving or bouncing along different paths within the visual field for 5 s in each trial; (b) animated figures of animals moving normally, also for 5 s; and (c) an optic-flow full-screen display with a field of dots moving back and forward and giving the impression of movement either toward or away from the viewer. All stimulus types contrasted with a static view of the same stimuli. The static views of the same stimuli shown in the dynamic, animated, condition constitute an optimal control condition, since all synesthetes strongly maintained that these stimuli did not evoke any synesthetic experience. All stimuli had a whitish background except the optical flow animation, which showed white dots on a black background. The visual angles for all presentation were 30 × 23 degrees, and the moving stimuli were not larger than two-three degrees. Participants viewed the visual stimuli through MR-compatible goggles, which were mounted to the head coil. E-prime software controlled the presentation of the visual stimuli and synchronization with the data acquisition of the MR scanner (E-Prime 2 Professional, Psychology Software Tools Inc.). The order of the three stimulus sets was intermixed.

In the first set of stimuli, each trial (5 s duration) showed a single geometrical object (triangles, squares, circles in different colors on a black screen) moving on a random path within the visual field. Animations were created by using PowerPoint, choosing two dimensional figures and animating them according to different paths (e.g., a circle bouncing like a ball). Each trial started and ended with a black screen. These trials were contrasted with trials of static presentations (4.5 s) of the same objects, followed by 500 ms black screen. The paradigm was a block design with six trials per block and in total six blocks per condition. Also, there were eight blocks with a fixation cross on a black screen. Each block for each condition lasted 30 s.

**FIGURE 1:**
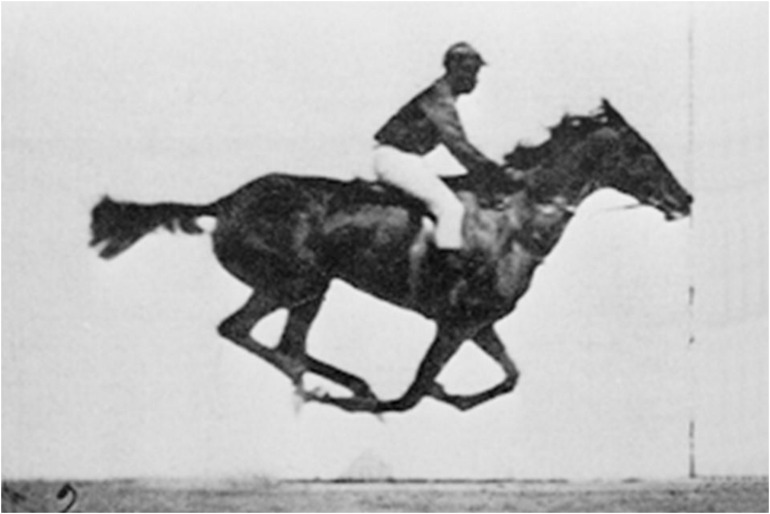
One still frame entitled “The horse in motion” (Eadweard Muybridge, considered to be the first animated film in history, 1878) from the soundless animations used in the MRI experiment.

The second set of stimuli consisted of animated movements of animals (see Figure 1 for a static example). Each trial lasted 5 s, and these trials were again contrasted with static views of the same animals. During the functional magnetic resonance imaging (fMRI) session, six blocks displayed movements, three blocks the static view, and three blocks the fixation cross, all arranged in a pseudo-randomized order and each block lasting 30 s.

The final stimulus set was an optic-flow display with a field of moving dots, contrasted with a static view of the same stimuli. Six blocks per condition were presented, and these blocks lasted only 18 s and alternated. This paradigm includes a black screen with a fixation cross only at the end of the animation. This paradigm allowed the online analysis of motion-sensitive brain areas on the MR scanner during data acquisition and served as navigator for the subsequent acquisition of the MR spectroscopy (MRS) data, as described below.

### Data Acquisition

The fMRI study used a 3-T GE Signa Exite scanner. The scanning protocol consisted of a high-resolution T1-weighted structural imaging, three fMRI runs, a DTI sequence (6 b0 images, 30 gradient directions), and concluded with four single-voxel MRS acquisitions (PRESS sequence, TE 35 ms, TR 1500 ms, 128 repetitions).

The axial slices for the functional imaging, based on an echo-planar imaging (EPI) sequence, were positioned parallel to the AC–PC line with reference to the structural image. The functional images were acquired with an EPI sequence, with the following parameter: 38 axial slices (128 × 128 matrix, 1.72 mm × 1.72 mm × 3.5 mm voxel size, field of view (FOV) 220 mm, echo time (TE) 30 ms, repetition time (TR) 3,000 ms, flip angle 90°). For the paradigm with the moving objects, 190 volumes were acquired, for the paradigm with the moving animals, 130 volumes were acquired, and the optic-flow paradigm consisted of 114 volumes.

### Data Analysis

#### Structural and Functional MRI Pre-processing and Analysis

The structural data were segmented using the VBM8 toolbox, which is an additional toolbox within the SPM framework (Mietchen and Gaser, 2009). The gray -matter maps of the segmented data were smoothed with a 6mm Gaussian kernel. Due to the skewed number of participants per group, the structural data were analyzed using the statistical non-parametric mapping (SnPM) toolbox (Nichols and Holmes, 2002), which is also an additional toolbox to the SPM framework. In addition, the total intracranial volume has been estimated and used as a regressor in the analysis. The analysis was performed with 1,330 permutations, and the SnPM results were explored with a cluster-inference statistic, using an initial voxel-threshold of *p* < 0.001 and a family wise error (FWE) corrected cluster threshold of *p*(FWE) < 0.05. As a confirmatory analysis, the VBM data were also analyzed as a series of three case-wise analyses by comparing each synesthete to the control group. The results from these three cases-wise analyses were explored as a global conjunction with a conservative *p*(FWE) < 0.05 threshold and at least 100 voxels per cluster. The global conjunction displays whether the effect is present in each synesthete, without the requirement of significance for each synesthete (Nichols et al., 2005).

The BOLD-fMRI data were pre-processed and statistically analyzed with SPM12.^1^ The EPI images were first realigned to adjust for head movements during the image acquisition, and the images were corrected for movement-induced distortions (“unwarping”). Data were subsequently inspected for residual movement artifacts. The realigned image series were then normalized to the stereotaxic Montreal Neurological Institute (MNI) reference space, provided by the SPM12 software package (using “Old Normalization”), and resampled with a voxel size of 2 mm × 2 mm × 2 mm. The images were then finally smoothed using a Gaussian kernel of 8 mm.

On the first level, the three stimulus sets were analyzed separately by specifying a general linear model (GLM) that contained the regressors for the respective conditions and the realignment parameter as covariates of no interest. Since all types of stimuli generated synesthetic sound experiences in the three synesthetes, the group analysis was conducted as a 2 × 3 analysis of variance (ANOVA) with the two factors groups (synesthetes/controls) and stimuli (animals/objects/optic-flow), and main effects, interactions, and mean effects were explored. The “movement – static” contrast images served as input to the ANOVA analysis. To gain reasonable protection against type-I errors, all analyses were examined with an FWE-corrected peak threshold of *p*(FWE) < 0.05, together with a cluster threshold of at least 10 voxels per cluster.

#### Analysis of Functional Connectivity

The results from the analysis of the structural data [Superior and Inferior Colliculus, MNI-coordinates (2 -24 -9)] were used as the seed region for analyses of the psycho-physiological interaction (PPI). Two PPI analyses have been performed. First, a regular PPI analysis was conducted that would highlight connection changes in response to the stimuli. However, it might also be the case that there is general difference in connectivity between the two groups, independent of the type of stimuli. Therefore, an additional PPI was conducted, using the “simple deconvolution” option of SPM12. In essence, this procedure identifies areas that show a correlated time course with the seed region. In the present study, the stimulus set regressors were not included in the PPI. Hence, this analysis showed a correlated activity that is present in both the movement and static conditions. Thereby, areas of intrinsic, stimulus-independent connectivity can be identified. All three fMRI paradigms were included in these analyses, and group effects were explored in the same way as outlined above. For both analyses, the area of significant activations were identified by using both the neuromorphic atlas that is implemented in SPM12 and using overlays on the AAL and Brodmann atlas included in the MRIcron software package. MRIcron has also been used for displaying the results.

## Results

### Structural Data

**FIGURE 2:**
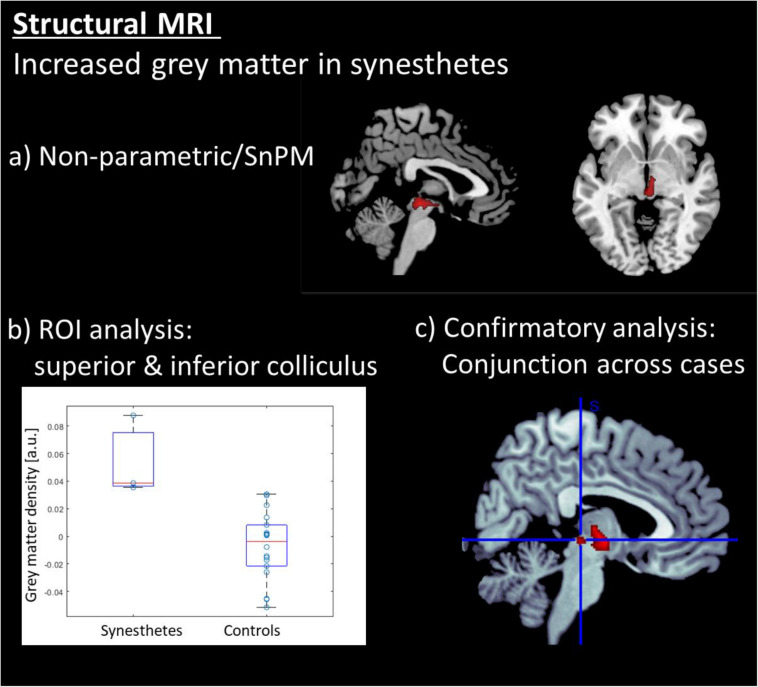
Results of the structural MRI analysis: **(a)** Voxel based morphometry (VBM) revealed that the synesthetes s have increased gray matter exclusively in the brainstem in an area comprising the inferior and superior colliculi. **(b)** ROI a analysis on the colliculi showing individual data points in grey matter density. **(c)** Conjunction analysis across the three synesthetes. All voxel-wise results are displayed at a corrected threshold of *p*(FWE) < 0.05.

**FIGURE 3:**
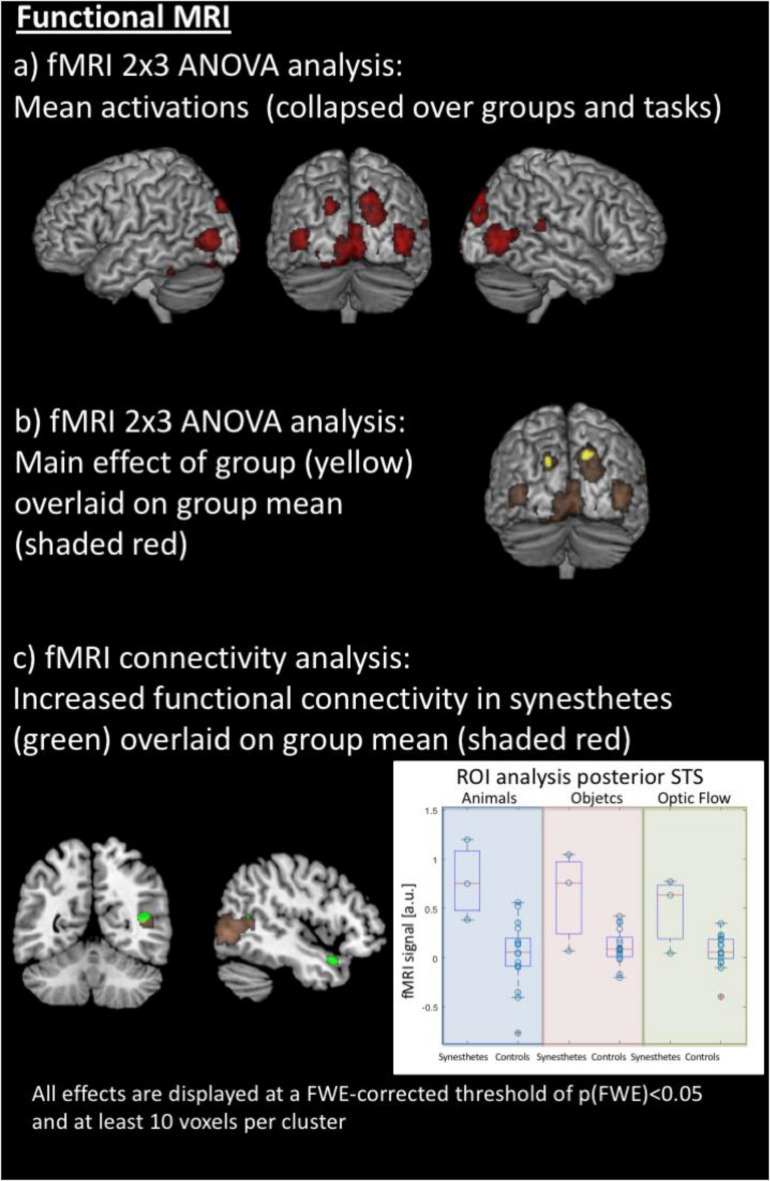
Results of the functional MRI analyses. **(a)** Averaged effect across groups and all stimuli in both hemispheres’ motion-sensitive regions of the posterior middle temporal gyrus, the cuneus and superior occipital gyrus, and the cerebellum. **(b)** Synesthetes show higher activation within the left and right cuneus, averaged over all stimuli, compared to the control group. **(c)** The inferior and superior colliculi as seed regions for functional connectivity analysis, using a simple deconvolution and correlation between the seed region and all other brain areas, reveal stronger functional connectivity for the synesthetes between the inferior and superior colliculi, the right posterior superior temporal sulcus (STS) and the right temporal pole; within the right STS, there is overlap of mean activations across groups, but a region of interest analysis (right panel) shows stronger activation for the synesthetes for all three stimulus sets (displayed in arbitrary units: a.u.).

The data were segmented with VBM8 and analyzed with a non-parametric test (SnPM), due to the small group size for synesthetes. Only the gray -matter maps were analyzed, and the results were explored at an FWE-corrected cluster threshold of *p*(FWE) < 0.05. The analysis revealed that the synesthetes have increased gray matter in the brainstem in an area comprising the inferior and superior colliculi (see Table 1 and Figure 2a). No other areas demonstrated an increased or decreased effect in gray matter. The individual data points (Figure 2b) indicates that there is no overlap in the distributions between the two groups. The results were further confirmed by a case-wise analysis and a conjunction across the three cases (Figure 2c). Specifically, the box plot in Figure 3c (as well as for the structural data in Figure 2c) show that the lower bound is one synesthete, the upper bound another, and the red line is the third.

**TABLE 1:** Results of VBM, fMRI, and PPI in the synesthetes and control groups (b) and synesthetes versus control groups (a, c, d).

Anatomy	Side	MNI coordinates			peak		cluster	
		*x*	*y*	*z*	T	*p*(FWE-corr)	size	*p*(FWE-corr)
**a) VBM: group difference (SnPM)**								
Brainstem/inferior and suprior colliculus	L/R	2	−24	−9	6.61	0, 3835	325	0, 0376
**b) fMRI: mean over conditions and groups**								
Posterior middle and inferior temporal gyrus, middle occipital gyrus	R	46	−72	0	10.94	<0.001	736	<0.001
Cuneus and superior occipital gyrus	R	16	−88	40	10.59	<0.001	417	< 0.001
Cuneus and superior occipital gyrus	L	−16	−86	32	9.32	<0.001	143	< 0.001
Lingual gyrus	R	14	−78	−8	8.54	<0.001	1106	< 0.001
posterior middle and inferior temporal gyrus, middle occipital gyrus	L	−44	−80	0	8.25	<0.001	336	< 0.001
Hippocampus	L	−24	−26	−12	6.68	0.001	22	0.001
posterior superior temporal gyrus	R	64	−34	18	6.34	0.002	59	<0.001
Cerebellum	L	−40	−50	−24	6.27	0.003	43	<0.001
Fusiform gyrus	L	−24	−80	−10	6.24	0.003	109	<0.001
Cerebellum	R	42	−46	−26	6.11	0.004	78	<0.001
Middle cingulate gyrus	R	14	−18	34	5.74	0.014	10	0.004
**c) fMRI: group difference**								
Cuneus	R	16	−88	38	7.65	<0.001	30	<0.001
Cuneus	L	−18	−86	34	6.47	0.001	19	0.001
**d) PPI: inferior and superior colliculus (group difference)**								
Posterior middle temporal gyrus	R	40	−56	12	8.34	0	24	0
Temporal pole	R	48	10	−20	7.16	0	23	0

### fMRI Data

The fMRI data were jointly analyzed in a 2 × 3 ANOVA with the factors of groups (synesthetes, controls) and paradigm (animals/objects/optic flow), with the contrast images between movement and the static view as input data. Results were explored at an FWE-corrected peak threshold of *p*(FWE) < 0.05, and at least 10 voxels per cluster. The averaged effect across groups and conditions revealed several areas activated by all three paradigms. These comprised regions of the posterior middle temporal gyrus at the border to the occipital lobe, the cuneus and superior occipital gyrus, and the cerebellum, in both hemispheres. In addition, the fusiform gyrus and hippocampus of the left hemisphere, and the posterior superior temporal gyrus and sulcus, lingual gyrus, and middle cingulate gyrus of the right hemisphere (see Table 1b and Figure 3a). The main effect of Groups showed that synesthetes had a higher activation to moving objects than the static view of the same objects, both within the left and right cuneus averaged over the three conditions (see Table 1c and Figure 3b).

Moreover, there was a main effect of Paradigm caused by stronger activations during the optic-flow paradigm. The effect was restricted to the primary and secondary visual cortex, and presumably caused by the different coverage of the visual field. Since differences between the paradigms are not the focus of this report and since there were no significant interaction effects, we did not examine further these paradigm-related effects.

### Functional Connectivity

The inferior and superior colliculi were seed regions for the analysis of the functional connectivity (PPI). The first PPI analysis explored whether the connectivity between the seed region and all other areas of the brain was modulated by the stimuli. Like described above, the effects were examined across the three sets. Results were explored at an FWE-corrected peak threshold of *p*(FWE) < 0.05. However, this analysis did not reveal any significant results, neither for the entire population nor as group contrast. Therefore, in a second PPI analysis, the hypothesis was tested whether the synesthetes may show a generally increased connectivity of the inferior and superior colliculi to other parts of the brain. This PPI analysis was conducted by using a simple deconvolution, which correlates the time-course of the seed regions with all other brain areas, thus highlighting stimulus-independent functional connectivity. Like described above, the effects were examined across the three sets, and, for simplicity, only differential group effects were examined – again with an *p*(FWE) < 0.05, and at least 10 voxels per cluster. The analysis revealed stronger and stimulus-independent functional connectivity for the synesthetes between the inferior and superior colliculi and the right posterior superior temporal sulcus (STS) and the right temporal pole (see Table 1d and Figure 3c, left panel).

Although the right posterior STS did not show a significant group difference in the voxel-by-voxel comparison of the brain responses to the three sets of stimuli (but a general activation, independent of the group, Figure 3a), a *post hoc* region of interest (ROI) analysis was performed for this area to test whether there was a difference in brain activation between synesthetes and control subjects, given the differential functional connectivity. This was done based on the hypothesis that the right posterior STS is considered as a multifunctional and multi-sensory area (Specht and Wigglesworth, 2018). Indeed, when analyzing the time courses for the posterior STS, using the peak voxel from the PPI analysis, the analysis indicated that the activation, averaged across the three sets, was significantly stronger in synesthetes than controls [*t*(18) = 4.0566, *p* < 0.0007] (see Figure 3c, right panel).

## Discussion

Structural MRI brain analyses revealed increased gray matter in motion-sound synesthetes in a brainstem’s cluster comprising the inferior and superior colliculi; notably, no other areas demonstrated an increased or decreased volume in gray matter (Figure 2). While both groups of participants observed passively the moving animations of objects, animals and an optical flow array, fMRI data showed activity in motion-sensitive regions, as well as temporal and occipital areas, and the cerebellum. However, the synesthetes had a higher activation within the left and right cuneus, with stronger activations during the optical flow stimulation. When the combined inferior and superior colliculi were used as a seed region for the analysis of the functional connectivity with all other brain areas, this revealed stronger functional connectivity for the synesthetes between the inferior and superior colliculi and the right posterior superior temporal sulcus and the right temporal pole (Figure 3).

We found not only enhanced volumes of the two subcortical nuclei in the synesthetes, compared to controls, but also increased connectivity between these subcortical areas and the posterior part of the superior temporal sulcus, close to the secondary auditory cortex. This area already been suggested in the original study on motion-sound synesthesia (Saenz and Koch, 2008b) as the cortical area most likely supporting the conscious percept of audio-visual integration (Specht and Wigglesworth, 2018). Hence, although no activation within the auditory cortex was detected, the ROI analysis indicated that synesthetes tended to show more activity in the adjacent multi-sensory posterior STS area. We note that auditory imagery in typical participants does not activate the primary auditory cortex, in contrast with visual imagery that is typically accompanied by above threshold activity in the visual system including primary sensory regions (e.g., Kosslyn et al., 2001). It should be emphasized that both groups showed activity in area STS (see Figure 3a), but only the synesthetes showed an increased connectivity of this area to the inferior and superior colliculi (see Figure 3c). The temporal pole too is considered a multimodal integration area, receiving visual and auditory input as well as visceral (e.g., smell and taste). This other temporal structure, though “enigmatic” in relation to its functions (Olson et al., 2007), could add semantic content to multisensory events and provide a link to emotional processes by binding visceral experiences. Hence, the present results provide an initial understanding into the integrative functions of these regions in the human brain, since the network outlined by the present structural and functional neuroimaging indicates not only binding in higher, multisensory, cortical areas but also in relation to subcortically audiovisual processing in the synesthetes’ brains. Notably, the increased connectivity appears to be independent of the sensory stimulation, since no modulations with the type of stimuli were detected.

### Enhanced Subcortical Connectivity in Synesthesia?

The enhanced structural connectivity account or cross activity hypothesis between local cortical neighbors has been able to explain successfully the two most frequent types of synesthesia (e.g., “calendar” and “grapheme-color” synesthesia). However, when seen in the light of the “small world” structural connectivity hypothesis, the occurrence of synesthesia of the motion-to-sound type might seems to require a different explanation (e.g., higher-level, long-range cortical, functional interactions). Nevertheless, we should not overlook the possibility that subcortical cross-activations between adjacent structures might constitute the relevant structural link for other types of synesthesia. Admittedly, many synesthetic inducers (e.g., musical features like chords and intervals) involve perceptual content of a high-level nature that makes it highly unlikely that their integration could occur at early, “low”, levels of sensory integration. However, we note that in the present case the acoustic and visual features reported by motion-sound synesthetes are clearly among the features processed in subcortical structures like the superior and inferior colliculi according to several animal studies.

In particular, the superior colliculus is able to track motion in visual space according to a gaze-based coordinate frame (Stein and Stanford, 2008). Notably, neurophysiological studies in animals (mainly the cat) suggest a fundamental role of the colliculi in combining spatial information across sensory modalities and controlling orienting behavior of sense organs (e.g., eyes) toward the stimuli’s locations regardless of their modality (Stein and Stanford, 2008). Thus, the colliculi detect and localize multisensory stimuli but their higher-level properties (e.g., object identity) must be represented in other, likely cortical, brain areas. Hence, the colliculi appear to provide a spatial indexing system in the mammalian brain that identifies multisensory signals that should be pooled together (Krauzlis et al., 2013), like the audio-visual integration of stimuli that move and make a sound concurrently (e.g., a buzzing insect; a person speaking, water falling or rushing in a river or a tap).

Moreover, the inferior colliculus tracks sounds within a tonotopic map and it is known to encode coarse spectral decompositions of complex sounds (Rodriguez et al., 2009), reacting to flux in timbre and sweeping or rhythmic fluctuations in pitch which are auditory features of several environmental sounds (objects sliding or bouncing). Interestingly, a fMRI study of musicians (Niranjan et al., 2019) revealed the involvement of the human inferior colliculus in processing spectrotemporal acoustic properties eminently represented by “activity” (roughness and flux from higher frequency bands) and “fullness” (fluctuations in lower frequency bands). Incidentally, these acoustic features correspond to the phenomenological “mickey mousing” of our synesthetes (e.g., the “whoosh” or “boink” sounds when seeing objects flying or bouncing).

Thus, as suggested by the known functionality of the superior colliculus in animals (Stein and Stanford, 2008), a synergy is obtained by combining visual and auditory information which results in an enhancement in detecting multisensory events in space and time. Descending excitatory inputs from multimodal areas of the cortex may, however, be necessary for the strongest expressions of multisensory integration to take place (e.g., “superadditive” effects; Stein and Stanford, 2008), at least in the cat. Regarding humans, Saenz and Koch (2008b) showed that sound-motion synesthetes were better in detecting the pulse of visual flashes, revealing that the synesthetic sounds, albeit illusory, allowed a better temporal resolution in visual perception (though no better than when auditory alone information was presented). Thus, the present results not only show how sound-motion synesthesia can be understood in relation to cross-modal functions within subcortical areas, but they may also provide a key to understand a likely underlying mechanism for the well documented synesthetic sensory advantages. Specifically, enhanced structural and functional connectivity between the colliculi of synesthetes and temporal lobe areas could make possible the integration of visual with (virtual) auditory information for a variety of spatial-temporal patterns, thus obtaining the typical benefit of cross-modal combinations.

### Limitations

The effects observed, though significant, were often of small size and one should be cautious since the chance for false positives is high with small sample sizes. Because the present report focuses on uncommon form of synesthesia, the sample size was necessarily smaller than samples in several studies of, for example, grapheme-color synesthesia (e.g., Rouw and Scholte, 2007). However, one of the values of the present MRI study is that it presents a “proof-of-concept” that cross-connectivity between adjacent regions at the subcortical level (e.g., in the colliculi) exists in synesthesia. In fact, even a single case can be highly informative and ripe with novel findings (e.g., Beeli et al., 2005) and previous studies with small groups of synesthetes have shown remarkably consistency with the studies with greater statistical power (see Rouw et al., 2011).

Other limitations were that (a) we used only a passive viewing “task”; an active task would have allowed us to relate performance levels with activity in the brain; (b) we used students for the control group, which was not optimally matched to all synesthetes, since one of the synesthetes was substantially older (40 y.o.) than the two others, contrary to the other two synesthetes who fell within the age range. Finally, we note that the colliculi are small sized regions and it is challenging to focus with MRI on individual differences, especially for the inferior and superior colliculi separately.

### Conclusion

Structural changes in the brains of synesthetes might be more widespread than expected (Bargary and Mitchell, 2008). The role of subcortical multisensory or multimodal links remains “terra incognita” in studies of humans in general. The present findings provide a way of implicating low-level mechanisms within the human neuroaxis as the substrate for local connectivity and cross activity between perceptual processes and modalities thought to be too “distant” from one another in terms of cortical topography to be accounted by structural changes.

## Data Availability Statement

The datasets presented in this study can be found in online repositories. The names of the repository/repositories and accession number(s) can be found below: The SPM result maps are deposited at https://identifiers.org/neurovault.collection:6169 (under the heading: Hearing motions).

## Ethics Statement

The studies involving human participants were reviewed and approved by The regional ethics committee of the State Department of Health (REK) in Norway approved the study (#2014/324). The patients/participants provided their written informed consent to participate in this study.

## Author Contributions

BL and KS conceived and designed the study. CF, KW, and AH recruited and tested the participants. KS performed the statistical analyses. BL wrote the first draft of the manuscript and all co-authors edited parts of the manuscript, read and approved the submitted version.

## Conflict of Interest

The authors declare that the research was conducted in the absence of any commercial or financial relationships that could be construed as a potential conflict of interest.

## Footnotes

1 http://www.fil.ion.ucl.ac.uk/spm
